# Transforaminal Epidural Injection for Far Lateral Lumbar Disc Herniations: An Alternative to Surgery or Just a Delay?

**DOI:** 10.7759/cureus.52530

**Published:** 2024-01-18

**Authors:** Luay Serifoglu, Mustafa U Etli

**Affiliations:** 1 Department of Neurosurgery, University of Health Sciences, Umraniye Training and Research Hospital, Istanbul, TUR

**Keywords:** radicular pain, non-surgical treatment, extreme lateral disc herniations, transforaminal epidural steroid injection, far lateral lumbar disc herniation

## Abstract

Objective: Far lateral lumbar disc herniations (FLLDH) are known for causing severe and persistent radicular pain due to direct nerve root and dorsal root ganglion compression. This study evaluates the effectiveness of transforaminal epidural steroid injection (TFSI) in patients with FLLDH, assessing its role as a potential alternative to surgery.

Methods: The study retrospectively analyzed 42 patients with radicular pain caused by FLDH, confirmed via lumbar magnetic resonance imaging, who had not benefited from conservative treatment. All patients underwent TFSI, and their pre-treatment Visual Analogue Scale (VAS) and Oswestry Disability Index (ODI) scores were compared with scores at one, two, and three months post-procedure.

Results: The study group comprised 19 males (45.23%) and 23 females (54.77%), with a mean age of 51.9 ± 11.63 years (range 29-76 years). The most common herniation levels were L4-L5 (22 patients), followed by L5-S1 (15 patients) and L3-L4 (five patients). The mean VAS score decreased significantly from 8.58 ± 0.63 to 2.89 ± 1.87 over three months (p = 0.001). Similarly, the mean ODI score significantly reduced from 61.29 ± 6.72 to 16.88 ± 11.25 (p = 0.001). However, eight of the 42 patients (19.04%) underwent surgery within three months due to lack of benefit from TFSI.

Conclusion: Our study sheds light on the significant potential of TFSI as a treatment option for FLLDH. The marked improvement in pain and functional capacity, as evidenced by the substantial decrease in VAS and ODI scores, suggests that TFSI can be an effective non-surgical intervention for a majority of patients suffering from FLLDH. However, a notable proportion of patients may still require surgery, indicating that TFSI might not be a definitive alternative but can be an effective interim treatment in managing FLLDH.

## Introduction

Far lateral lumbar disc herniations (FLLDH) are a distinct and challenging subset of lumbar disc herniations, constituting approximately 3-11% of lumbar disc herniation cases [1]. Magnetic resonance imaging is the best diagnostic imaging modality for FLLDH [2]. Characterized by the extraforaminal position neighboring the exiting nerve, FLLDH differs from the more prevalent central and paramedian herniations, primarily more severe in standing position and persistence of the radicular radiating leg pain that is caused by continuous compression [3]. This pain is attributed to the direct compression of the nerve root and dorsal root ganglion, often leading to significant clinical impairment with leg pain that limits movements and a reduction in the quality of life for affected individuals [4].

The management of FLLDH has traditionally been a complex endeavour within spinal care. When conservative treatments fail, surgical intervention could be a choice with many techniques that have developed with studies like midline or paramedian surgery, tubular approach, and endoscopic technique [5-9]. These approaches are limited due to various patient-related factors such as overall health, age, or specific anatomical considerations [10,11]. Consequently, there is a need for less invasive strategies with similar efficiency profiles to reduce tissue harm, stay in the hospital period, and make patients return to their work or normal day life earlier [12].

In this context, Transforaminal Epidural Steroid Injection (TFSI) has strong evidence as a potential non-surgical treatment option for lumbar disc herniations. TFSI involves precisely delivering corticosteroids and anaesthetics into the epidural space near the affected nerve root.

The primary goal of this procedure is to reduce inflammation and alleviate pain. While the efficacy of TFSI is well-documented for central and paramedian herniations, its effectiveness in treating FLLDH remains under-explored and ambiguous [1]. This study aims to fill this critical gap in knowledge by rigorously evaluating the impact of TFSI on patients with FLLDH. Focusing on critical outcomes such as pain reduction, functional improvement, and the potential need for subsequent surgical interventions, the study seeks to ascertain whether TFSI can be considered a viable alternative to surgery in managing FLLDH.

## Materials and methods

This retrospective study was designed to investigate FLLDH patients at Umraniye Training and Research Hospital (Istanbul, Turkey) between 2022 and 2024. We evaluated 42 patients diagnosed with FLLDH. The inclusion criteria were adult patients with single-level FLLDH detected on Magnetic Resonance Imaging(MRI) and supported with neurological examination. All patients with FLLDH had radiating leg pain compatible with the side of herniation and did not respond to conservative treatments. After neurological examination, the patients noted the Visual Analogue Scale (VAS) score of their pain as 0 represented 'no pain' and 10 indicated 'the worst imaginable pain. They were also questioned on the Oswestry Disability Index (ODI) questionnaire and scores were recorded. The ODI questionnaire includes 10 items covering various aspects such as intensity of pain, personal care, lifting, walking, sitting, standing, sleep quality, social life, travelling ability, and changes in pain. All patients included in the study had at least three months of follow-up.

We excluded patients with disc herniations other than FLLDH, spinal infections, spondylolisthesis, neoplasms, multilevel disc herniations, spinal stenosis, and a history of lumbar surgery or injections. We also excluded patients under the age of 18 years and adult patients with the missing information in the file archive. We declared a detailed explanation of the procedure to all participants and they signed informed consent. All patient information was taken from the hospital digital file archive.

TFSI procedure

The TFSI procedure was performed by a single surgeon under strict monitoring of vital parameters. Patients were positioned prone, and the lumbar area was sterilized with an antiseptic solution. The level of the procedure was determined using C-arm fluoroscopy (Siemens, Germany). After obtaining anterior-posterior images, the C-arm was positioned obliquely at 15-20 degrees to localize the foramen. Local anaesthesia was administered using 1 mg of 1% lidocaine. A 22-gauge 90 mm lumbar puncture needle was then used to access the skin and subcutaneous tissues, directing the tip towards the intervertebral foramen. The correct needle positioning in the foramen was confirmed with serial lateral and oblique X-rays. Following this, 0.5 cc of radio-opaque solution was injected to confirm the typical ventral epidural spread of the contrast agent. Once adequate dispersion was established, a mixture of 40 mg (1 mL) methylprednisolone acetate (Depo-Medrol, Pfizer Pharmaceuticals Ltd, Luleburgaz, Turkey) and 10 mg (2 ml) bupivacaine hydrochloride (Marcain 0.5%, Astra Zeneca, Istanbul, Turkey) was injected into the foramen. The procedure was performed slowly and carefully. Post-procedure, patients were monitored for four to six hours in the recovery room for any early complications before discharge.

Statistical analysis

Descriptive statistical data such as mean, standard deviation, minimum, maximum, and median were used for continuous variables. Chi-squared test used in categorical datasThe paired t-test was employed to compare changes in pain levels before and after the injection. A p-value of less than 0.05 was considered statistically significant. All statistical analyses were performed using SPSS Statistics version 23 (IBM Corp., Armonk, NY).

## Results

Demographic and clinical characteristics

The study encompassed a diverse group of 42 patients, with ages ranging from 29 to 76 years and a mean age of 51.9 ± 11.63 years. The gender distribution was relatively balanced, with 19 males (45.23%) and 23 females (54.77%). The distribution of herniation levels among the patients was as follows: 22 patients had herniations at the L4-L5 level, 15 at the L5-S1 level, and five at the L3-L4 level. The demographic data and the spinal levels of FLLDH are shown in Table 1.

**Table 1:** Demographics of patients and levels of far lateral lumbar disc herniations

Patient Demographics	Values mean
Age(year)	51.9 ± 11.63 (range 29-76)
Gender	Values number(%)
Male	19(45.23%)
Female	23(54.77%)
Levels of Disc Herniations	Values number(%)
L3-4	5(11,90%)
L4-5	22(52,38%)
L5-S1	15(35,71%)

This distribution highlights the prevalence of FLDH in the lower lumbar spine. There was no statistically significant difference between the demographic data and the initial VAS and ODI scores of the patients, indicating a uniform baseline across the cohort.

**Figure 1:**
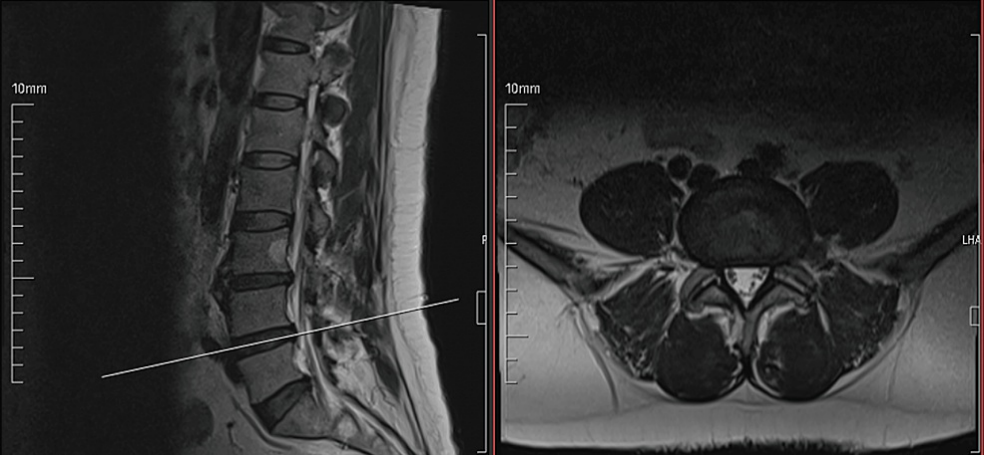
Magnetic resonance images Sagittal (left-hand side image) and axial (right-hand side image) images of left-sided far lateral lumbar disc herniation at the L4-5 level.

**Figure 2:**
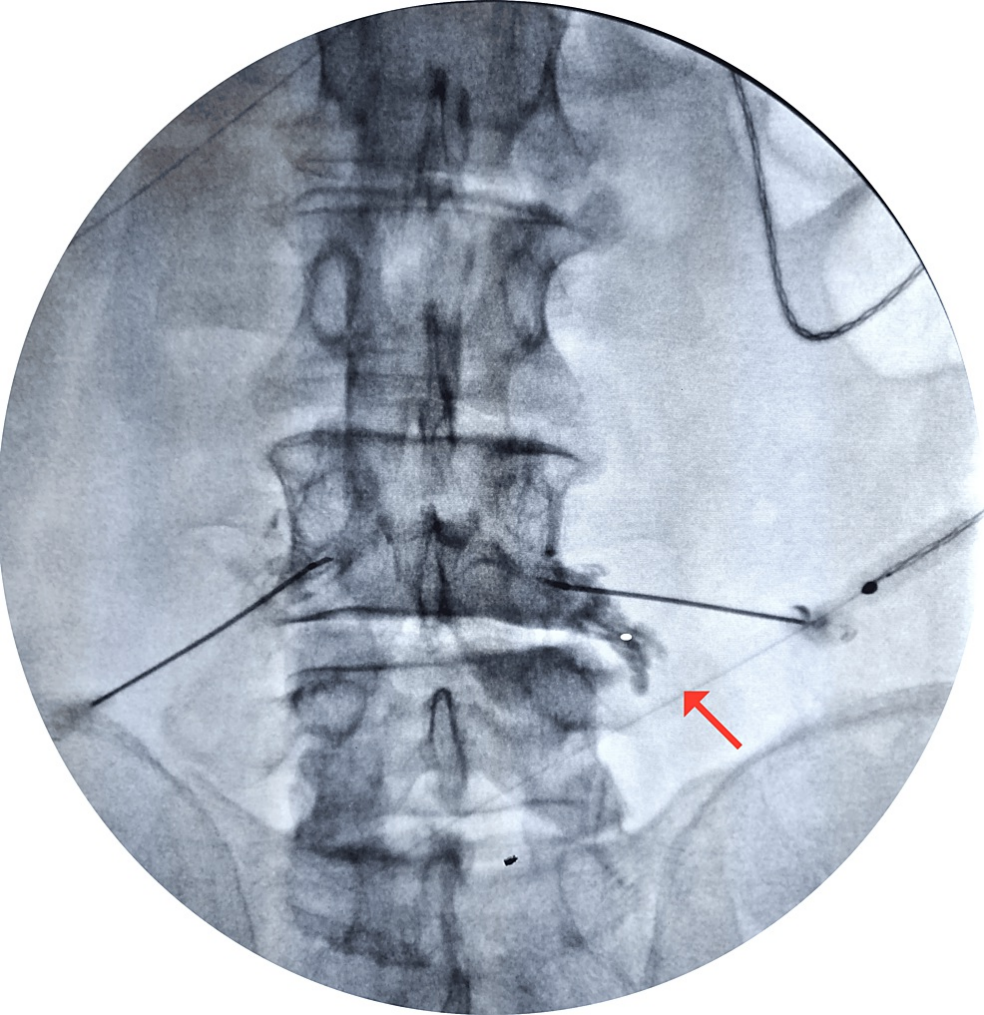
Transforaminal epidural steroid injection technique Transforaminal epidural steroid injection technique under floroscopy with contrast agent confirmation is shown with the red arrow.

The patients with FLLDH (Figure 1) were treated with TFSI (Figure 2) and then the effectiveness of TFSI was primarily measured through changes in VAS and ODI scores over three months.

The mean VAS score at the outset was 8.58 ± 0.63, indicating severe pain. This score decreased significantly to 4.13 ± 1.35 in the first month, 3.89 ± 0.79 in the second month, and further to 2.89 ± 1.87 in the third month. The reduction in VAS scores over time was statistically significant (p = 0.001), demonstrating the effectiveness of TFSI in alleviating pain.

Similarly, the ODI scores, which reflect the impact of pain on daily life and activities, showed a significant decrease. The baseline mean ODI score was 61.29 ± 6.72, indicating a substantial disability. This score reduced to 35.49 ± 3.96 in the first month, 29.21 ± 11.2 in the second month, and further to 16.88 ± 11.25 in the third month. The decrease in ODI scores was also found to be statistically significant (p = 0.001), suggesting an improvement in the functional status of the patients. The VAS and ODI values and comparisons are shown in Table 2.

**Table 2:** VAS and ODI scores of patients Pre-procedure of transforaminal injection and post-procedural first, second, and third-month Visual Analog Scale (VAS) and Oswestry Disability Index (ODI) scores of patients.

	Pre-procedure	1st month	2nd month	3rd month	p value
VAS	8.58 ± 0.63	4.13 ± 1.35	3.89 ± 0.79	2.89 ± 1.87	0.001
ODI	61.29 ± 6.72	35.49 ± 3.96	29.21 ± 11.2	16.88 ± 11.25	0.001

Despite the overall positive response to TFSI, eight out of the 42 patients (19.04%) underwent surgical intervention within three months following TFSI due to insufficient pain relief. Among these, two patients had herniations at the L4-L5 level, and six were at the L5-S1 level. This subset of patients represents those for whom TFSI did not provide the necessary relief, indicating the variability in response to this treatment modality.

## Discussion

The results of our study offer a comprehensive understanding of the role of TFSI in managing FLLDH. The significant reduction in both VAS and ODI scores over three months post-TFSI is a testament to the potential of this intervention in effectively reducing pain and enhancing functional capacity in patients with FLLDH. This aligns with existing literature on the benefits of C-arm or computed tomography-assisted TFSI in addressing radicular pain associated with lumbar disc herniation, reinforcing its position as a valuable treatment modality [13,14]. If the conservative and interventional methods like TFSI fail the surgery should be done [15]. The surgical methods, especially transforaminal endoscopic discectomy, are favorable because of minimal invasiveness and direct access to the far lateral disc herniation anatomical area [16].

Evran and Katar [17] investigated 37 patients with FLLDH and concluded that TFSI is an effective method for gaining increased functional capacity and pain control in the treatment of patients who are not suitable for surgical treatment with radicular complaints due to far lateral lumbar disc hernia similar to our results.

Erhard et al. [18] investigated a case in which a patient with FLLDH treated with TFSI reported a significant decrease in both ODI and pain rate. That patient returned to running on alternate days for a minimum of 30 minutes, which was the patient's primary goal. ODI and VAS scores were like our results [18].

Kim et al. [19] evaluated 15 and 70 patients in the FLLDH and intraspinal herniation of lumbar disc groups with fluoroscopically guided TFSI. They found significant improvement in the VAS and ODI score demonstrated in the FLLDH group 12 weeks after injection; moreover, they concluded that there was no statistically significant difference in the VAS and ODI between both the FLLDH and intraspinal disc group [19].

Cha et al. [20] investigate the caudal epidural steroid injection for lumbar disc herniation at different locations, such as central, foraminal and extraforaminal. They concluded that centrally located disc herniations had good outcomes from caudal epidural steroid injections [20]. They had a small number of extraforaminal disc herniations and none of them had a good outcome. It could be the anatomical area.

The study's findings particularly underscore the viability of TFSI as an alternative to surgical intervention for FLLDH. With approximately 80.96% of patients experiencing substantial relief, TFSI emerges as a compelling first-line treatment option, especially considering the inherent risks and recovery associated with spinal surgery. Given the complexity and potential complications of surgical procedures in the lumbar spine, this is a crucial consideration.

Zhao et al. [21] examined the clinical outcomes of lumbar transforaminal epidural injection between ultrasound and fluoroscopy guidance for lumbar radiculopathy, and they found that ultrasound-guided lumbar transforaminal injection confirmed by fluoroscopy was not inferior to conventional fluoroscopy method in terms of accurate rate of lumbar epidural contrast dispersion [21]. Also, they concluded that effective pain relief and functional ability improvement were comparable between the two modalities, and the ultrasound technique had the advantages of less radiation exposure and possible facilitation of avoiding critical vessels around the intervertebral foramen [21].

However, it's essential to acknowledge that TFSI did not prove effective for all patients in the study. About 19.04% of the participants did not find adequate relief from TFSI and subsequently underwent surgical procedures. This variation in response highlights the need for a personalized approach to treating FLLDH. Factors such as the severity of symptoms, patient-specific anatomical considerations, and overall health must be carefully evaluated to determine the most appropriate treatment course [22].

The study has limitations because of its retrospective nature. The small, limited sample size may affect the broader applicability of the findings. Additionally, the absence of a control group treated with alternative methods or a placebo limits the ability to draw more definitive conclusions about the efficacy of TFSI compared to other treatments. Future research endeavours, ideally incorporating larger patient groups, randomized controlled trials, and extended follow-up durations, would be invaluable in further clarifying the effectiveness of TFSI in FLLDH management.

In summary, our study reinforces the notion that TFSI is a promising non-surgical treatment for FLLDH, capable of providing significant pain relief and functional improvement for most patients. While not universally successful, it offers a potential alternative to surgery, meriting consideration in the treatment plan for patients with FLLDH. The findings highlight the importance of tailored treatment strategies and underscore the need for ongoing research to optimize therapeutic approaches for this patient population.

## Conclusions

In conclusion, our study sheds light on the significant potential of TFSI as a treatment option for FLLDH. The marked improvement in pain and functional capacity, as evidenced by the substantial decrease in VAS and ODI scores, suggests that TFSI can be an effective non-surgical intervention for a majority of patients suffering from FLLDH. However, a notable proportion of patients may still require surgery, indicating that TFSI might not be a definitive alternative but can be an effective interim treatment in managing FLLDH if supported with more datas.
